# Food availability modulates the combined effects of ocean acidification and warming on fish growth

**DOI:** 10.1038/s41598-020-58846-2

**Published:** 2020-02-11

**Authors:** Louise Cominassi, Marta Moyano, Guy Claireaux, Sarah Howald, Felix C. Mark, José-Luis Zambonino-Infante, Myron A. Peck

**Affiliations:** 1 Institute of Marine Ecosystem and Fisheries Science, Center for Earth System Research and Sustainability (CEN), University of Hamburg, 22767 Hamburg, Germany; 2 Université de Bretagne Occidentale, LEMAR (UMR 6539), Centre Ifremer de Bretagne, 29280 Plouzané, France; 3 Alfred Wegener Institute Helmholtz Centre for Polar and Marine Research, Integrative Ecophysiology, 27570 Bremerhaven, Germany; 4 Ifremer, LEMAR (UMR 6539), Laboratory of Adaptation, Reproduction and Nutrition of Fish, Centre Ifremer de Bretagne, 29280 Plouzané, France

**Keywords:** Feeding behaviour, Ecophysiology

## Abstract

When organisms are unable to feed *ad libitum* they may be more susceptible to negative effects of environmental stressors such as ocean acidification and warming (OAW). We reared sea bass (*Dicentrarchus labrax*) at 15 or 20 °C and at ambient or high *P*CO_2_ (650 versus 1750 µatm *P*CO_2_; pH = 8.1 or 7.6) at *ad libitum* feeding and observed no discernible effect of *P*CO_2_ on the size-at-age of juveniles after 277 (20 °C) and 367 (15 °C) days. Feeding trials were then conducted including a restricted ration (25% *ad libitum*). At 15 °C, growth rate increased with ration but was unaffected by *P*CO_2._ At 20 °C, acidification and warming acted antagonistically and low feeding level enhanced *P*CO_2_ effects. Differences in growth were not merely a consequence of lower food intake but also linked to changes in digestive efficiency. The specific activity of digestive enzymes (amylase, trypsin, phosphatase alkaline and aminopeptidase N) at 20 °C was lower at the higher *P*CO_2_ level. Our study highlights the importance of incorporating restricted feeding into experimental designs examining OAW and suggests that *ad libitum* feeding used in the majority of the studies to date may not have been suitable to detect impacts of ecological significance.

## Introduction

An amalgam of abiotic and biotic factors interact in nature to impact the vital rates of marine organisms^1–3^ and understanding the cumulative effect of multiple stressors on marine organisms is currently one of the top priorities for ecologists^4^. Unfortunately, the effect of multiple stressors is challenging to predict because their interaction can be either additive (the combined response is the sum of responses to individual factors), synergistic (the combined response is greater than the sum of responses to the independent factors) or antagonistic (the combined response is smaller than the response to either single)^5^. For example, the projected increase of the concentration of carbon dioxide (CO_2_) in the atmosphere by 2100 (from 280–410 ppm to 730–1020 ppm^6,7^) is expected to cause both ocean acidification (OA, decrease in pH by 0.3 to 0.5 units^6^) and continued global warming (0.2 °C increase per decade in the past 30 years^8^). Research efforts are underway to understand how OAW will combine to impact on the vital rates of marine biota^9,10^.

When consumers in marine food webs have been exposed to OAW, a range of changes affecting growth responses have been reported^11^. The level of OAW projected for 2100 caused significant reductions in the growth of mollusks and echinoderms, but a variety of responses has been reported in fish. For example, larval sea bass (*Dicentrarchus labrax*) incubated in four treatment groups (17 and 19 °C, 600 and 1000 µatm *P*CO_2_) grew significantly faster in the warmer and higher *P*CO_2_ treatment^12^ whereas growth of Senegalese sole (*Solea senegalensis*) larvae increased with temperature but decreased with increasing *P*CO_2_^13^. A main conclusion to be drawn from the mixed results reported for fish is that the effect of OA and OAW can be life stage- and species-specific, and warming could either offset or aggravate any impacts of OA^14^. An important caveat is that the vast majority of studies investigating the effects of OA or OAW, on fish or other marine animals, has been performed on individuals fed *ad libitum*. *Ad libitum* rations may provide ample energy allowing organisms to compensate for potential negative impacts of sub-optimal levels of temperature and/or *P*CO_2_ on energy acquisition, dissipation and allocation. For instance, invertebrates such as corals, mussels and oysters maintained on restricted rations displayed more deleterious effects to OAW than well-fed conspecifics^15–18^. In fish, only very recent studies have examined the influence of the interaction between CO_2_ and food ration on larval growth and development^19–22^.They showed either no supplementary effect with food restriction^19,20^ or observed larger individuals but with important organ damages^22^.

Covering obligatory maintenance costs (standard metabolic rate) is generally the first priority when organisms allocate available energy. When additional food resources are available, however, the corresponding energy allocation to discretionary activities is based on fine-tuned trade-offs that depend on the organisms’ activities, physiological state and environment. For instance, during long-term food restriction, energy is not available to fuel the production of digestive enzymes which inevitably impairs digestive capacity and reduces rates of growth and protein synthesis in fish^23^. Environmental changes might also impact energy allocated for digestion and consequently for growth. For example, 15 months after European sea bass larvae were exposed to an 8-day hypoxic episode, their growth rates and protein digestive capacity (lower trypsin activity in the pancreas and aminopeptidase N and alkaline activity in the intestine) were still lower than those from siblings maintained in normoxia^24^. Information on how OA will impact the digestive function of marine organisms is relatively scarce^25^. The hypothesis is that OA will act as a metabolic stressor, similar to hypoxia, causing reduced digestive capacity. If OA impaired acid base regulation, more energy might be allocated to buttress this homeostasis (or others defense mechanisms) at the cost of digestive efficiency. Although there is no evidence yet that digestive function might be affected, Strobel *et al*.^26^ demonstrated that in an exposure of an Antarctic fish to 2000 µatm *P*CO_2_, regulation of acid-base balance occurred at the detriment of other processes such as calcification or osmoregulation likely due to changes in energy allocation.

We examined the growth rate and digestive capacity of juvenile sea bass (*Dicentrarchus labrax*) fed *ad libitum* or restricted (25% of *ad libitum*) rations at an ambient and an elevated (+1100 µatm) level of *P*CO_2_. Two trials were conducted using juveniles that had been reared for nearly a year under OA conditions since the early larval stage. The first trial was performed on fish reared at 20 °C while the second trial was conducted about 2 months later on fish reared at 15 °C. The time between trials allowed the 15 °C fish to grow to a body size more comparable to that of the warm-acclimated fish at the start of the first trial. We focused on understanding the underlying mechanisms of potential impacts of OA on growth including feed conversion efficiency (FCE), stomach pH and the activity of key digestive enzymes. Although OA had no discernable impact on size-at-age of sea bass feeding at *ad libitum*, expectations were that elevated *P*CO_2_ combined with restricted feeding would cause decrements in growth performance in these fish, particularly at the warmer temperature. Incorporating feeding level treatments in a long-term exposure to OAW, this study reveals that the elevated temperature and elevated *P*CO_2_ levels acted antagonistically on juvenile fish growth and highlights the need to re-examine the design of experiments attempting to test “real world” effects of climate-driven changes in abiotic factors.

## Results

### Growth performance

**Figure 1:**
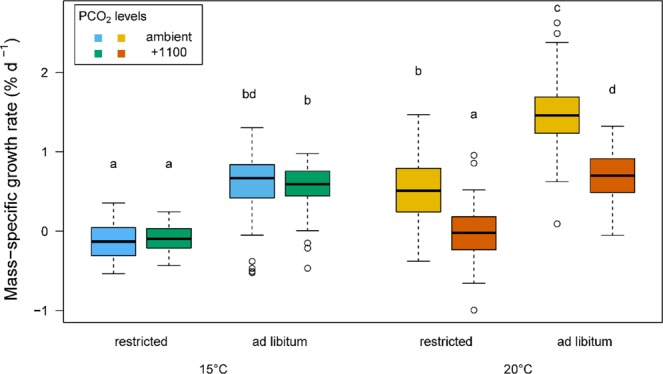
Box and whisker plots of mass-specific growth rate (SGR) of individually tagged juveniles sea bass (n = 103 to 115 per box) reared at two temperatures (15 and 20 °C), two *P*CO_2_ levels and two feeding levels^94^. Different letters denote significant differences (Student-Newman-Keuls test, p < 0.05) between each condition. The whiskers denote the 10^th^ and 90^th^ percentiles, the box denotes the 25^th^ and 75^th^ percentiles, the median value is shown (horizontal line) as well as outliers (points).

Prior to the trial, for each temperature treatment, no significant differences were observed in the mass-at-age of fish reared since larvae at different *P*CO_2_ levels (measurements conducted at 277 dph and 367 dph for 20 °C and 15 °C, respectively; see Fig. S2). No mortalities occurred during the trials and individual SGR (specific growth rate) ranged from −0.53 to 1.30% d^−1^ at 15 °C and from −0.99 to 2.62% d^−1^ at 20 °C (Fig. 1). Growth appeared to be similar across all body sizes, from relatively small to large fish at 15 °C (S3) and 20 °C (S4). At 15 °C, SGR was not affected by *P*CO_2_ and, not unexpectedly, fish fed *ad libitum* grew significantly faster than those fed restricted rations (ANOVA, p < 0.001). A different pattern emerged at 20 °C, where SGR was significantly affected not only by ration level but also by *P*CO_2_, as indicated by a significant interaction (ANOVA, p < 0.001). For fish fed *ad libitum*, the SGR of ambient fish was 110% higher than fish from the elevated (+1100 µatm) treatment. In restricted feeding condition, the difference was even more pronounced, as the +1100-acclimated fish lost mass, while the groups of fish reared at ambient *P*CO_2_ had a mean SGR of 0.5% d^−1^.

**Figure 2:**
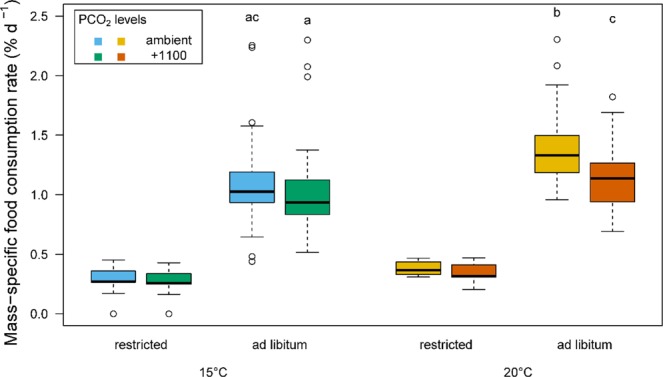
Box and whisker plots of the mass-specific food consumption rate at two temperatures (15 and 20 °C,) two *P*CO_2_ levels and two feeding levels (n = 103 to 115 per box)^94^. Differences in specific food consumption rate were tested between *ad libitum* groups. Different letters denote significant differences (nested ANOVA, p < 0.05). The whiskers denote the 10^th^ and 90^th^ percentiles, the box denotes the 25^th^ and 75^th^ percentiles, the median value is shown (horizontal line) as well as outliers (points).

For *ad libitum* fed fish, the temperature (ANOVA, p < 0.001), the *P*CO_2_ level (ANOVA, p < 0.001) and the interaction between the two (ANOVA, p = 0.004) significantly affected the specific food consumption rate. At 15 °C, the specific food consumption rate was similar between the two *P*CO_2_ levels (Student-Newman-Keuls multiple comparison test, p = 0.051), while at 20 °C the specific food consumption rate was higher in the ambient (1.38(0.04) % d^−1^) compared to the +1100 *P*CO_2_ (1.12(0.03) % d^−1^) treatment group (Fig. 2). Importantly, the specific food consumption rate was similar between fish at 15 °C in the ambient treatment and those at 20 °C in the +1100 *P*CO_2_ treatment (Student-Newman-Keuls multiple comparison test, p = 0.285). The day-to-day feeding patterns of fish in the *ad libitum* treatments were variable and lacked any regular periodicity (Fig. S5). The total daily food intake at 20 °C, however, was lower in the ambient compared to the +1100 *P*CO_2_ treatment on 13 of the 18 days (nested ANOVA, p < 0.001, Fig. S5).

**Figure 3:**
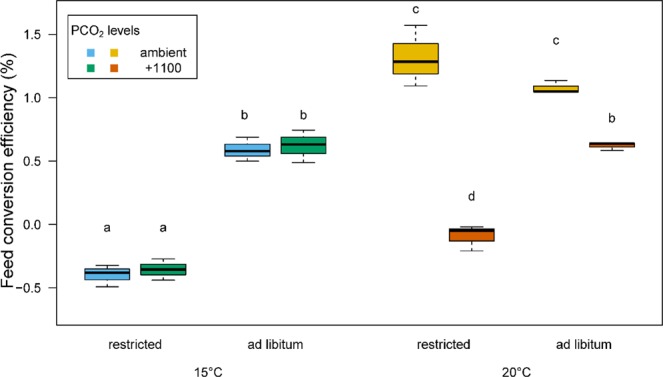
Box and whisker plots of feed conversion efficiency (FCE) of juvenile sea bass reared at two temperatures, two *P*CO_2_ levels and at two feeding levels (n = 3 mean, tank values per box)^94^. Different letters denote significant differences (Student-Newman-Keuls test, p < 0.05) between each treatment group. The whiskers denote the 10^th^ and 90^th^ percentiles, the box denotes the 25^th^ and 75^th^ percentiles and the median value is shown (horizontal line).

The FCE (feed conversion efficiency) was highest for fish in the ambient *P*CO_2_ treatment at 20 °C (>1.0) and was reduced by almost half (<0.6) in fish at 20 °C in the +1100 treatment. At 20 °C and ambient *P*CO_2_, the FCE of fish fed restricted and *ad libitum* rations was not significantly different. In contrast, FCE in fish in the +1100 and restricted ration treatment was negative at both temperatures (mean(± SE); −0.35(0.08) and −0.09(0.10) for 15 and 20 °C, respectively). The level of *P*CO_2_ had a significant effect on FCE at 20 °C (ANOVA, p < 0.001) but not at 15 °C. The largest difference in FCE between the ambient and +1100 *P*CO_2_ treatments was observed at both feeding levels at 20 °C (Fig. 3).

### Kinetics of stomach pH

**Figure 4:**
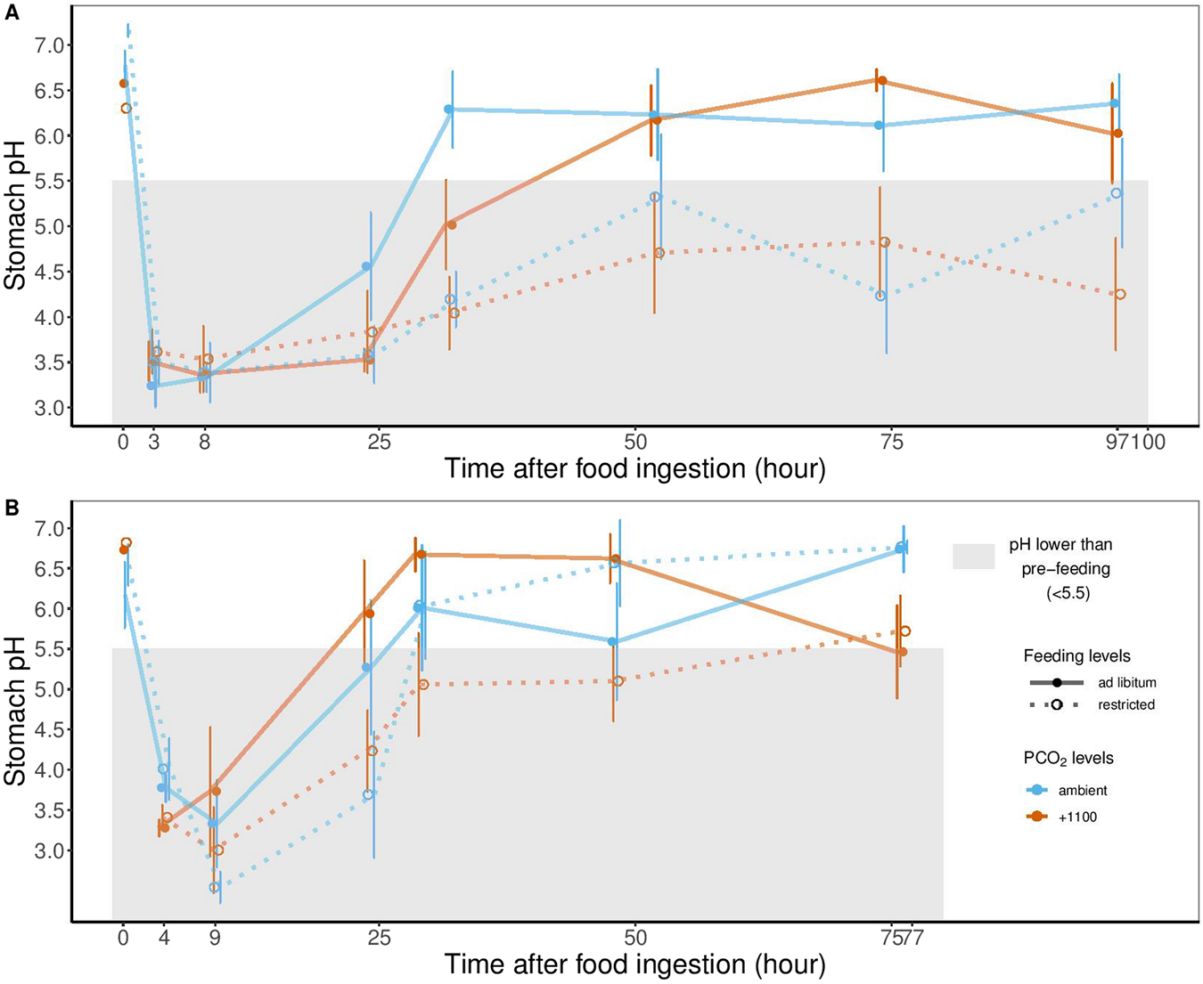
Post-prandial kinetics of stomach pH in juvenile sea bass^94^. Symbols display the mean(± SE, n = 8) for fish reared at two *P*CO_2_ levels (colors) and two feeding levels (shape) at either (**A**) 15 °C or (**B**) 20 °C. In both panels, the lines are model predictions (LME).

Prior to feeding, mean(± SE) stomach pH ranged from 6.17(0.40) to 7.15(0.07) and was similar at both temperatures, *P*CO_2_ levels and feeding levels. In all treatments, acid was rapidly secreted after feeding and pH declined. The lowest pH values were measured at the first, two post-prandial sampling times (between 3 and 9 hrs post-feeding). Stomach pH after 3 (15 °C) and 4 (20 °C) hrs post feeding was similar among treatments (Kruskal-Wallis, p = 0.719 and p = 0.117 for 15 and 20 °C, respectively), as well as after 8 (15 °C) and 9 (20 °C) hrs post-feeding (Kruskal-Wallis, p = 0.996 and p = 0.725 for 15 and 20 °C, respectively). After this initial decrease, the time course of stomach pH depended on the rearing treatment. At 15 °C, the time needed for stomach pH to return above a standard value (5.5) was significantly affected by feeding level (ANOVA, p < 0.001). The return was slower for fish fed restricted feeding levels (Fig. 4). At 20 °C, the kinetics of stomach pH of fish from the restricted feeding +1100 µatm treatment were significantly different (Tukey, p = 0.034) compared to the *ad libitum* one. Fish fed restricted rations at +1100 µatm took nearly twice as long to return to pre-feeding pH values (e.g. 48 versus 26 hrs).

### Enzyme measurement

**Figure 5:**
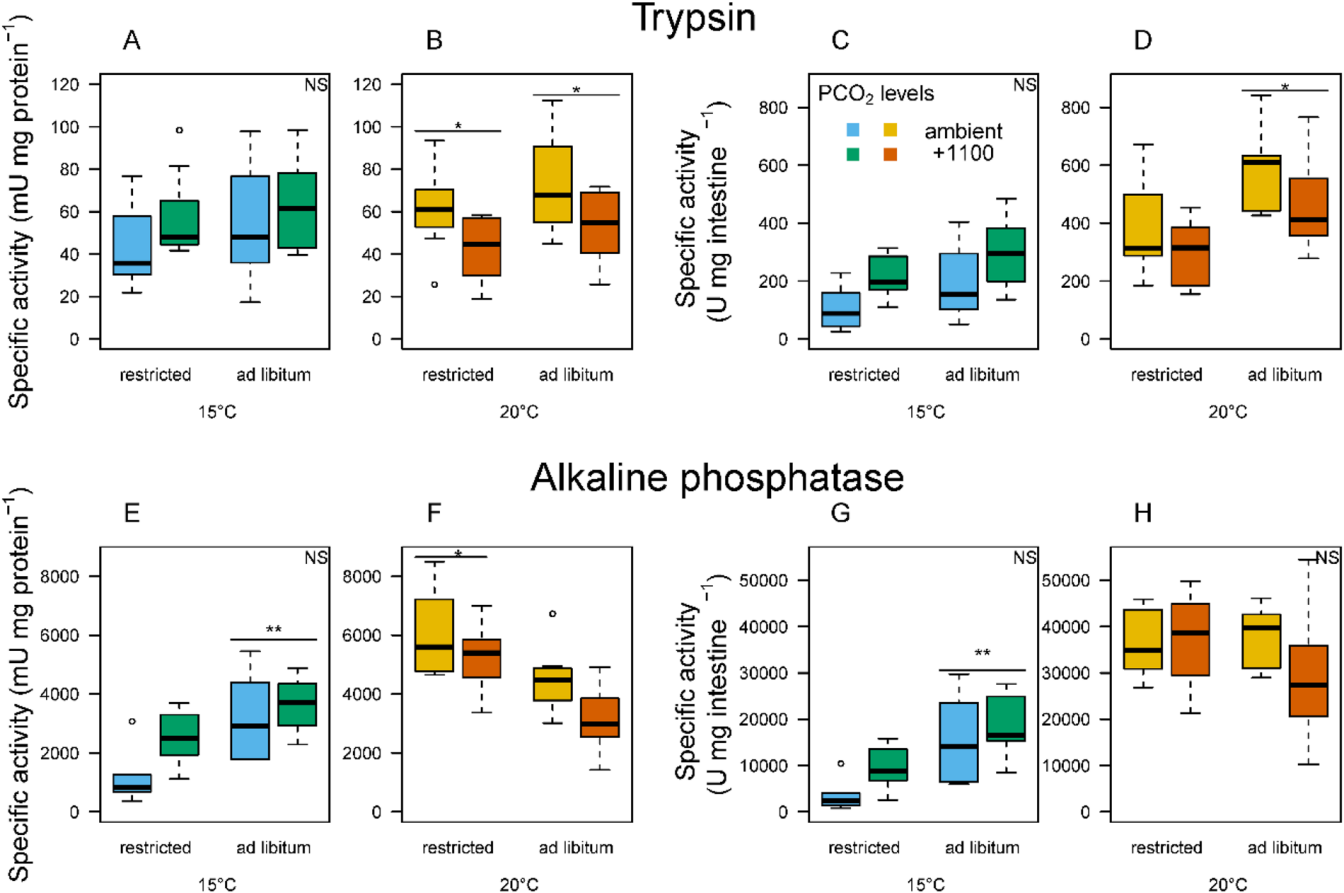
Box and whisker plots (n = 8) of specific activities of digestive enzymes of fish reared at two temperatures according to *P*CO_2_ levels and feeding levels^94^. Results are given in milli-units per mg of protein (mU mg protein^−1^) for specific activity and in units per mg of intestine segment (U mg intestine^−1^) for total activity. Stars denote significant differences (ANOVAs, * < 0.01, ** < 0.001). The whiskers denote the 10^th^ and 90^th^ percentiles, the box denotes the 25^th^ and 75^th^ percentiles, the median value is shown (horizontal line) as well as outliers (points).

The total activity of each of the four tested enzymes was significantly lower at the colder compared to the warmer temperature (ANOVA, p < 0.001). Total enzyme activity also tended to be higher in fish fed *ad libitum* versus restricted rations (Fig. 5). At 15 °C, the total activity of AP (alkaline phosphatase) was significantly higher for fish on the *ad libitum* versus the restricted ration (ANOVA, p < 0.001; Fig. 5G). At 20 °C, the total activity of trypsin was significantly higher for fish on the *ad libitum* versus restricted rations (ANOVA, p = 0.005) (Fig. 5D).

Significant reductions in the specific activities of AP (ANOVA, p < 0.001) and aminopeptidase N (ANOVA, p = 0.007) were observed in fish at 15 °C compared to 20 °C. At 15 °C, AP was significantly lower in fish fed restricted versus *ad libitum* rations (ANOVA, p < 0.001; Fig. 5E). In contrast, at 20 °C the specific activity of AP was higher in fish fed restricted rations (Fig. 5F). At 15 °C, despite a tendency for the specific activity of all four enzymes to be higher at the high versus the ambient *P*CO_2_ level, no significant differences were found (ANOVA, p > 0.05) (Fig. 5). At 20 °C, in contrast, the specific activity of AP tended to decline with increasing *P*CO_2_ and that for trypsin was significantly lower at high *P*CO_2_ (ANOVA, p = 0.009). Indeed, the specific activity of trypsin in fish at +1100 *P*CO_2_ at 20 °C was ≈70% that of fish in the ambient treatment (Fig. 5B).

## Discussion

Knowledge on the combined effect of multiple stressors on the ecology of species and communities is still relatively scarce^4,27^ and there remains an urgent need to conduct experiments incorporating such interactions. Ocean acidification and warming (OAW) appears to induce a variety of responses in well-fed marine organisms^9^. The results of the present study suggest that different responses may have been observed in those studies if restricted feeding levels had been employed. In the present study, although no differences were observed in sea bass growth after nearly one year of rearing under OAW, obvious and significant differences in various aspects of growth physiology were observed due to OA when restricted feeding levels were applied in the feeding-growth trials.

Although the effect of OA on food intake and growth of marine organisms appears to be taxon-, species- and life stage-specific, the results of the growing number of studies suggest that potentially deleterious effects of OA may be offset if ample food is available^28,29^. In calcifying organisms, for instance, it has been shown that food supply modulated the impact of low pH on growth^17,28,29^. Along that line, work on the eastern oyster (*Crassostrea virginica*) indicated that food availability partially offset the impacts of OA on larval growth and development^16^. Similarly, examination of the growth dynamics of polyps of two jellyfish species suggested that potential climate-driven changes in prey stoichiometry were more important in determining mass-gain than OA per se^30^. On the contrary, no differences were observed in the growth rate and skeletal development of cod (*Gadus morhua*) larvae reared at high CO_2_ and provided either a low or high food concentration^22^. To maintain similar rates of growth, however, those authors suggested that compensatory energy (re-)allocation occurred since larvae reared at low prey concentrations also displayed organ damage, especially to the liver. Although the growth performance of fish, particularly juveniles, is considered to be largely unaffected under high levels of *P*CO_2_^31,32^, energy limitation imposed by restricted feeding may cause increased sensitivity to environmental stressors^22,33^.

Prior to the feeding trial, no difference in growth was observed regarding *P*CO_2_ treatment, while after the trial a difference in growth was observed between ambient and +1100 *P*CO_2_, at 20 °C. During long-term rearing, growth measurements were made on a subsample of fish from each tank while, in contrast, growth information was available for each (individual tagged) fish during the feeding trial. Moreover, while no mortalities occurred during the trials, ~40% mortality occurred during rearing after larvae were transferred to juvenile tanks. European sea bass are known to be cannibalistic and it is possible that a larger number of small individuals were eaten in the +1100 *P*CO_2_ treatment during long-term rearing and cannibalism would have biased (increased) the growth rates calculated for surviving fish in this treatment^34^. The mean growth rate of *ad libitum*-fed individuals in this study was 1.4% d^−1^ at 20 °C which is similar to growth rates (0.6 to 1.5% d^−1^) reported for sea bass reared at comparable feeding levels and temperature^35,36^. Given that optimal temperatures for growth in juvenile sea bass were reported to be 22 to 24 °C^37^, faster growth was expected at 20 °C compared to 15 °C in the present study. Although this was the case for fish in the ambient *P*CO_2_ treatment, mean growth rates of well-fed fish were similar at 20 and 15 °C in the high *P*CO_2_ treatment.

The individuals used in the present study originated from wild-caught adults acclimatized and reared (~5 years) in an aquaculture facility. The adults spawned at 13 °C at a pH of 7.6 corresponding to our elevated condition of acidification. Adults were maintained in running seawater (a flow-through system) and not in a recirculating aquaculture system in which *P*CO_2_ levels could be relatively high. Previous studies have reported that the sensitivity of offspring to acidification might decrease with parental conditioning to high *P*CO_2_^38–40^. Therefore, the absence of differences in growth observed here at 15 °C might be a consequence of transgenerational plasticity (TGP). This potential TGP, however, was absent at 20 °C. We are unaware of studies suggesting that TGP is expressed in only a narrow range of parental temperatures and OA conditions. Griffith and Gobler (2017)^41^ highlighted that TGP is likely species-specific and, to our best knowledge, no studies have investigated TGP in sea bass.

Previous studies have indicated that *P*CO_2_ may alter rates of feeding^42,43^. For example, feeding and foraging activities of juvenile anemonefish (*Amphiprion melanopus*) were depressed at moderate levels of *P*CO_2_ (530 µatm) but enhanced at a higher level of *P*CO_2_ (960 µatm)^42^. On the contrary, reduced feeding by Chilean abalone (*Concholepas concholepas*) larvae at high *P*CO_2_ were reported^43^. Similarly, in the present study, daily *ad libitum* feeding rate was lower in fish in the high *P*CO_2_ treatment at 20 °C (but not 15 °C) compared to the normocapnic treatment. Although not quantifyied, it was also clear that fish fed restricted level of food swam much less than the *ad libitum* fed fish (personal observation). Differences in SGR (specific growth rate) also existed between ambient and high *P*CO_2_ in fish maintained at a similar restricted ration level, suggesting that differences in growth were driven by differences in feed conversion efficiency (FCE) and not food consumption rate. The FCE depends on several, interacting factors such as feeding level and water temperature^44–46^. The relatively low FCE at 15 °C (i.e. ~ 0.5) further highlights that this is a sub-optimal temperature for the growth of juvenile sea bass. The highest feed efficiency, however, occurs when fish are fed slightly below satiation^47^, which agrees with the pattern observed between the two ration levels at 20 °C and ambient *P*CO_2_. At 20 °C and high *P*CO_2_, a drastic reduction in FCE was observed for fish on restricted rations compared to fish fed *ad libitum*. It is possible that high *P*CO_2_ increases maintenance or activity costs and/or reduces digestive capacity, and consequently reduces growth. Substantial levels of feeding providing sufficient energy income, however, could counteract those effects.

Values and changes in gastric pH as functions of stomach fullness and digestive stage have been well characterized in fish^48–50^. Although some teleosts constantly secrete acid to maintain low stomach pH, even in the absence of food^51^, other teleosts, such as sea bass, only secrete acid in response to food ingestion. As a result, pH values in empty stomachs are generally less acidic (>pH 5.5) in these species^52^. Depending on the time since feeding, post-prandial pH values between 1.8 and 5.5 were measured in this study, which agrees well with values reported in a previous study on the same species^53^. Post-prandial changes in stomach pH observed in the present study were also similar to those reported for other temperate and sub-tropical teleosts such as gilthead sea bream (*Sparus  aurata*) and the white sea bream (*Diplodus sargus*)^48,54^, but were faster than those reported in a previous study on larger sea bass at 26 °C^53^. After 3 or 4 hrs post-feeding, there was no significant difference in stomach pH across the feeding and *P*CO_2_ treatment groups with mean(± SE) values between 3.24(0.23) and 3.62(0.24) at 15 and 20 °C, respectively. This initial decrease in stomach pH, due to the strong production of hydrochloric acid by the gastric glands, was sufficient to allow maximum activity of important proteases in the stomach (e.g. activity of pepsin is maximal at pH values of 1.5 to 4.0^54^). There were treatment-specific differences in the time needed for pH values to return to pre-feeding (> 5.5) levels. The post-prandial return of stomach pH was influenced by both meal size and *P*CO_2_. Ingesting large amounts of food leads to larger stomach distension^53^ that promotes stronger and more frequent peristaltic contraction and increased rates of food evacuation^55^. Results showed that, except for 20 °C- *ad libitum* fish, high *P*CO_2_ led to a slower post-prandial return of stomach pH to more neutral, pre-feeding levels. Based on changes in oxygen consumption rate, a similar prolongation in digestion time was observed in Atlantic cod exposed to elevated CO_2_^56^. Tirsgaard *et al*.^56^ assumed that an extended digestion time and slower stomach clearance might lower food intake. In fish fed *ad libitum* at 20 °C, this slow return of stomach pH to pre-fed levels was observed in fish in the +1100 µatm *P*CO_2_ treatment but not for fish in the ambient treatment, which may explain why the latter group consumed more food than the former group. As the alimentary bolus enters the intestine, a rapid buffering takes place through intense bicarbonate secretion into the intestine lumen^57^. We did not examine the time course of this process but it would be interesting to do so given the contribution of bicarbonate to maintaining acid-base homeostasis under hypercapnic conditions.

Higher values of enzyme activity were expected when feeding fish *ad libitum* rations. Surprisingly, at 20 °C, the specific activity of AP was higher when animals were feed-restricted. The potential preservation or increase in AP activity under dietary restriction is, to our knowledge, a unique finding in fish but similar results were reported in mice where restricted energy intake led to a significant increase in intestinal AP^58^. A second unexpected finding was the lack of a significant effect of feeding level (at both temperatures) on the specific activities of amylase and aminopeptidase N. A lower activity of both enzymes was expected with feed restriction^59,60^. The reason for this response is unknown.

Recent studies have shown that exposure to high *P*CO_2_ can trigger an increase in the secretion of intestinal bicarbonate (HCO_3_^−^)^61^ which, in turn, enhances AP activity^62^. In normocapnia, bicarbonate is secreted in the proximal intestine in response to low intestinal pH^63^. This secretion makes the intestine more alkaline, bringing pH closer to the optimum value for enzyme activity, such as AP^64,65^. Such regulation of HCO_3_^−^ secretion in response to low intestinal pH would explain why there was a tendency for higher enzymes activities in fish from the +1100 compared to the ambient *P*CO_2_ treatment at 15 °C. This tendency, however, was absent when individuals were reared at a warmer (+5 °C) temperature. Indeed, antagonistic effects (*P*CO_2_ × temperature) were found for trypsin and AP activity. Thus, enzymatic activities were reduced at +1100 compared to ambient *P*CO_2_. Similar antagonistic patterns in digestive enzymes have been reported in newly born bamboo sharks (*Chiloscyllium punctatum*) experiencing OAW (+4 °C x ~1400 µatm *P*CO_2_)^66^.

The decrease in the specific activity of AP and trypsin under hypercapnia observed in the present study are similar to those (AP) reported in Senegalese sole (*Solea senegalensis*) larvae^67^. Several studies have suggested that reduced activity of trypsin is an important mechanism limiting growth rate^68,69^. Trypsin is necessary for protein hydrolysis but the production of this enzyme requires the activation of machinery for cellular protein production. This activation represents another energetic demand^70^ in addition to the energy already invested to exchange ions and release bicarbonate. This additional energetic cost may be difficult to cover for fish persistently experiencing restricted feeding conditions. Such reductions in trypsin activity have been observed in juvenile sea bass exposed to hypoxia early in life^24^. It must be noted, however, that we measured the specific activity of trypsin in the lumen of the intestine, and that measurements in the pancreas would be necessary to verify this hypothesis. Moreover, reductions in digestive capacity could also result from direct damage to gut tissues as was demonstrated in young Atlantic cod reared under high *P*CO_2_ conditions^71^.

Overall, using a long-term exposure to OAW throughout the first year of life and examining the mechanisms of growth performance in a marine fish, the present study demonstrates how high feeding levels can reduce the impact of OAW. High *P*CO_2_ reduced the growth of juvenile sea bass reared at 20 °C and these effects were exacerbated in fish fed restricted rations. Reduction in growth was not merely due to reduced food intake but also related to processes decreasing feed conversion efficiency such as digestive capacity (e.g. via reductions in the specific activity of digestive enzymes). Many of these deleterious impacts of *P*CO_2_ on sea bass were not observed at 15 °C, a sub-optimal temperature for the growth of juveniles of this species. In this study, the focus was on the impact of OAW combined with food availability. It is important to note that nutritional requirements of sea bass may differ under OA compared to ambient (present-day) conditions; consequently, fish on restricted rations may have not only experienced decreased caloric/energy intake but also an additional impact of poor nutrition. Changes in global nutritional requirements under OA would be interesting to examine in the context of energy allocation and digestive efficiency. Our study emphasizes the need to integrate different, ecologically relevant feeding levels in laboratory experiments assessing effect of OAW on marine organisms and suggests that previous studies that have used *ad libitum* feeding may underestimated the deleterious impacts of OAW.

## Materials and Methods

The present work was performed within Ifremer-Centre de Bretagne facilities (agreement number: B29-212-05). Experiments were conducted according to the ethics and guideline of the French law and legislated by the local ethics committee (Comité d’Ethique Finistérien en Experimentation Animal, CEFEA, registering code C2EA-74) (Authorization APAFIS 4341.03, permit number 2016120211505680.v3).

### Animals and experimental conditions

#### Water parameters

Sea bass used in the present experiments were reared since 3 days post-hatch (dph), under one of 4 different OAW treatments including two different *P*CO_2_ levels (ambient and high (+1100)) and two thermal treatments (15 °C and 20 °C). The ambient *P*CO_2_ was approx. 650 µatm. This is equal to today’s situation for coastal waters of Brittany (Cameron and Iwama, 1987; Pimentel *et al*.^33^) where, in 2014, the annual mean *P*CO_2_ level was 603 µatm (range 284–888 µatm) in the Bay of Brest (Salt *et al*. 2016^7^). The IPCC Representative Concentration Pathway (RCP) 8.5 scenario projected an increase of ~500 µatm above current values by the end of the century (IPCC, 2014^7^). The *P*CO_2_ level in coastal areas and estuaries, habitats where sea bass juveniles and adults are encountered, however, is much higher^72,73^). In these shallow water coastal systems, *P*CO_2_ levels often above 2000 µatm have been reported^74,75^. In accordance with these and additional *P*CO_2_ levels in European estuaries reported by Frankignoulle *et al*.^76^, the second treatment was fixed at +1100 µatm above the ambient level (labelled +1100, approx. 1700 µatm). The 15 °C treatment included larval rearing at 15 °C while juveniles experienced naturally fluctuating thermal conditions between 15 and 18 °C (natural, seasonal differences reflecting ambient summer conditions in the Bay of Brest^77,78^, http://marc.ifremer.fr/en/results/temperatureand_salinity/mars3dchannel_bay_of_biscay_mode/(typevisu)/map/(zoneid)/sudbzh#appTop]). The 20 °C treatment included larval rearing at 20 °C while juveniles experienced 20 to 23 °C (5 °C increase relative to ambient temperature). The 5 °C increased was defined based of the ‘business-as-usual’ (RCP 8.5) scenario as predicted by the Global Climate Models (GCMs) by 2100 (IPCC, 2007^79^). Constant temperature were applied for the larval stage which experience relatively stable temperature offshore while juveniles reach the estuaries in the late spring and are then exposed to seasonal change in temperature^80,81^.

Sea water was pumped in from the Bay of Brest from a depth of 20 m approximately 500 m from the coastline, passed through a sand filter (~500 µm), heated (tungsten, Plate Heat Exchanger, Vicarb, Sweden), degassed using a column, filtered using a 2 µm membrane and finally UV sterilized (PZ50, 75 W, Ocene, France) assuring high water quality. Replicate treatment tanks (n = 3 for larval rearing and n = 2 for juveniles rearing) were supplied with sea water via header tanks where water *P*CO_2_ was controlled using IKS Aquastar system (IKS Computer Systeme GmbH, Germany). This system continuously measured water pH and was equipped with a solenoid valve that regulated the flow of CO_2_ from the gas cylinder using feedback from the pH electrode. The valve was turned on and off according to the electrode measurement. This valve, therefore, controlled the amount of CO_2_ injected in the water flowing through the header tank into the fish rearing tank (flow rate: 0.18 L min^−1^, corresponding to a water exchange of 30% per hour). Temperature and pH were checked ((WTW 3110 pH meter, Xylem Analytics Germany, Weilheim, Germany; with electrode: WTW Sentix 41, NIST scale) each morning before feeding. The pH meter as well as the IKS Aquastar system were calibrated daily with NIST certified WTW technical buffers pH 4.01 and pH 7.00 (Xylem Analytics Germany, Weilheim, Germany). Total alkalinity was measured once a week following the protocol of Anderson & Robinson^82^ and Strickland & Parsons^83^: 50 ml of filtered tank water (200 µm nylon mesh) were mixed with 15 ml HCl (0.01 M) and pH was measured immediately. Total alkalinity was then calculated with the following formula:

$$TA=\,\frac{{V}_{HCl}\cdot {c}_{HCl}}{{V}_{sample}}-\frac{({V}_{HCl}+{V}_{sample})}{{V}_{sample}}\cdot \frac{\{{H}^{+}\}}{{\gamma }_{{H}^{+}}}[\frac{mol}{l}]$$

With: TA – total alkalinity [mol * l^−1^], V_HCl_ – volume HCl [l], c_HCl_ – concentration HCl [mol * l^−1^], V_sample_ – volume of sample [l], H^+^ – hydrogen activity (10^-pH^), γ^H+^ – hydrogen activity coefficient (here γ^H+^ = 0.758).

The Microsoft Excel macro CO2sys^84^ was used to calculate seawater carbonate chemistry, the constants after Mehrbach *et al*.^85^ (as cited in CO2sys) refit by Dickson & Millero^86^, were employed. Using the CO2sys, daily pH (NIST) values were c converted to pH (free) values. Oxygen saturation (WTW Oxi 340, Xylem Analytics Germany, Weilheim, Germany) and salinity (WTW LF325, Xylem Analytics Germany, Weilheim, Germany) were measured once a week together with total alkalinity, from juvenile stage onwards, see all water parameters in Table 1.

**Table 1:** Water parameters during the larval and juvenile phase of batch 2016: Larval period at 15 °C (L 15 °C) from 3 to 60 dph and 20 °C (L 20 °C) from 3 to 46 dph.

Treatment	pH [free scale]	Temp. [°C]	Salinity [psu]	O2 [% airsat.]	TA [mol l^−1^]	*P*CO_2_ [µatm]
L 15 °C A	7.95 ± 0.01	15.3 ± 0.0	33.0 ± 0.1	—	2364 ± 17	656 ± 16
L 15 °C +1100	7.58 ± 0.00	15.3 ± 0.0	33.0 ± 0.1	—	2394 ± 26	1682 ± 26
L 20 °C A	7.88 ± 0.01	20.0 ± 0.1	33.1 ± 0.1	—	2369 ± 21	832 ± 13
L 20 °C +1100	7.60 ± 0.01	20.0 ± 0.1	33.1 ± 0.1	—	2380 ± 23	1672 ± 33
J 15 °C A	7.97 ± 0.01	16.0 ± 0.2	34.2 ± 0.1	90.9 ± 0.5	2396 ± 18	655 ± 18
J 15°C +1100	7.55 ± 0.01	16.1 ± 0.2	34.2 ± 0.1	90.9 ± 0.6	2399 ± 19	1841 ± 40
J 20 °C A	7.92 ± 0.01	21.9 ± 0.2	35.0 ± 0.2	90.2 ± 0.9	2418 ± 12	788 ± 22
J 20 °C +1100	7.59 ± 0.01	21.9 ± 0.2	35.0 ± 0.2	91.3 ± 0.6	2423 ± 12	1808 ± 65
SW 15 °C	8.05 ± 0.01	14.5 ± 0.5	33.0 ± 0.2	101.2 ± 0.6	2434 ± 21	522 ± 18
SW 20 °C	7.95 ± 0.02	21.2 ± 0.4	32.7 ± 0.1	102.3 ± 1.4	2433 ± 28	723 ± 33

Juvenile period at 15 °C (J 15 °C) from 61 to 387 dph and 20°C (J 20 °C) from 47 to 280 dph. Mean(± SE) values are provided over all replicate tanks per treatment. Temperature (Temp.) and pH (NIST) were measured daily and converted to pH (free). Salinity, total alkalinity (TA) and oxygen concentration (O2, juveniles only) were measured weekly and *P*CO_2_ was calculated with CO2sys. Sea water (SW) measurements were conducted during 2017 and 2018. A = Ambient *P*CO_2_, +1100 = ambient + 1100 µatm *P*CO_2_.

### Larval and juvenile rearing

Larvae used in this experiment were the progeny of wild brood stock fish caught off Morbihan, France, and kept at an aquaculture facility (Aquastream, Ploemeur-Lorient, France). Four females (mean mass 4.5 kg) were crossed with ten males (mean mass 2.4 kg), which spawned naturally using photothermal manipulation. At 2 dph, larvae were transferred to the Ifremer-Centre de Bretagne. Larval rearing was performed in a temperature-controlled room using black, 35-L tanks. Rapid acclimation to a new temperature (e.g. to 20 °C), feeding regime and photoperiod was implemented as described by Gourtay *et al*.^87^ until the juvenile stage. Juvenile were moved to 670-L tanks at 50 dph and 65 dph for fish reared at 20 °C and 15 °C, respectively. There were randomly allocated to two treatment tanks. Having only two replicates limited our ability to estimate variation but dividing the fish randomly will remove any potential tank effect during larval rearing. Prior to trials, during the rearing of juveniles, mortality was between 24.8 and 43.4% per tank. Juveniles were fed *ad libitum* daily rations of commercial fish food (Neo Start, Le Gouessant, Lamballe, France) using automatic feeders. Photoperiod was adjusted to natural conditions once a week. The tanks were cleaned daily after pH-measurements. Water flow rates maintained oxygen saturation levels above 90%.

### Feeding-growth trial

At 8 and 11 months post-hatch, for the 20 °C and the 15 °C rearing condition, respectively, fish between 10 and 100 g were selected for the feeding trials (about 90% of all juveniles). Fish were subcutaneously tagged (Passive integrated transponder; Pit-tag) for individual identification and randomly allocated among 12 indoor, 500-L tanks supplied with filtered and aerated natural seawater. Fish were excluded that i) were < 10 g since these were too small to be tagged, ii) had any morphological deformities, and iii) were> 100 g. Fish were allocated (maintaining *P*CO_2_ history) so that there was a similar mean and variance of fish sizes and, hence, similar total biomass in each replicate tank (mean ± SE; 1876.72 ± 30.94 g (~33 fish) and 1287.30 ± 14.87 g (~35 fish), for 20 °C and 15 °C trials, respectively). Feeding-growth trials commenced after a> 7-day acclimation period to the tanks (Fig. S1). Juveniles were 303 and 399 dph at the start of the 20 °C and 15 °C trials, respectively, and had a mean(± SE) wet mass of 52.13 (0.62) and 31.08 (0.42) g, respectively. Three replicate tanks for each *P*CO_2_ treatment were randomly assigned to *ad libitum* and “restricted” feeding treatments. Feed was administrated during daylight hours. In the *ad libitum* treatment, fish were fed three times a day (at 09:00, 13:00 and 17:00). A known initial mass of food (30 and 50 g for 15 and 20 °C fish, respectively) was partially distributed to each tank three times a day (09:00, 13:00 and 17:00). Food was delivered by hand making sure that no food was left uneaten. The mass of food not distributed to each tank was determined. The mass fed (consumed by fish) was the difference between the final and initial masses of feed for a tank on that day. The mean value for the three replicate *ad libitum* tanks was determined and 25% of that value was set as the ration for the restricted feeding group the next day (starting at 9:00 and distributed using an automatic feeder). The restricted ration was fed using an automatic feeder starting at 9:00. Food consumption of *ad libitum*-fed fish showed daily variation (reported in the Fig. S5). The 20 and 15 °C trial lasted 18 and 38 days, respectively.

At the start and end of the trial, every fish was slightly anesthetized with tricaine methanesulfonate (MS-222; dose adapted to water temperature and fish mass, typically 0.2 g l^−1^) and wet mass (WM) was measured (Cubis MSE12201S-000-D0, Sartorius, Germany; d = 0.1 g). Specific growth rate (SGR, % d^−1^) and feed conversion efficiency (FCE, %) were calculated according to the following formulas:

$${\rm{SGR}}=100(\mathrm{ln}[{{\rm{WM}}}_{{\rm{final}}}]-\mathrm{ln}[{{\rm{WM}}}_{{\rm{initial}}}])/{\rm{Number}}\,{\rm{of}}\,{\rm{Days}}\,{\rm{of}}\,{\rm{Feeding}}$$

$${\rm{F}}{\rm{C}}{\rm{E}}={\rm{B}}{\rm{i}}{\rm{o}}{\rm{m}}{\rm{a}}{\rm{s}}{\rm{s}}{\rm{g}}{\rm{a}}{\rm{i}}{\rm{n}}\,({\rm{g}})/{\rm{T}}{\rm{o}}{\rm{t}}{\rm{a}}{\rm{l}}\,{\rm{M}}{\rm{a}}{\rm{s}}{\rm{s}}\,{\rm{o}}{\rm{f}}\,{\rm{F}}{\rm{o}}{\rm{o}}{\rm{d}}\,{\rm{C}}{\rm{o}}{\rm{n}}{\rm{s}}{\rm{u}}{\rm{m}}{\rm{e}}{\rm{d}}\,{\rm{i}}{\rm{n}}\,{\rm{t}}{\rm{h}}{\rm{e}}\,{\rm{T}}{\rm{a}}{\rm{n}}{\rm{k}}\,({\rm{g}})$$

With Biomassgain corresponding to the final wet biomass minus initial wet biomass in the tank.

### Determination of digestive enzymes

Fish were sampled for digestive enzymes twice, once one day before the start of the trial (after the acclimation period) and one week after final weighing, while keeping them on the two rations levels (experimental day 29 at 20 °C, and 49 at 15 °C, see Fig. S1). Fish were starved for 48 h prior to both samplings and each time 8 to 9 individuals were randomly sampled per treatment. Fish were dissected on ice, the abdominal cavity was opened and the intestine was separated from the rest of the gut. For each fish, the mucosa of the digestive track was collected by scraping the anterior of the intestine, put directly in 1.5-ml microtubes and stored at −80 °C. To purify brush border membranes, intestinal mucosa was homogenized according to a method described by Crane *et al*.^88^. This included homogenizing the intestinal mucosa for 20 s (ultra turax, Poltron PT2100, Kinematica AG, Switzerland) at maximum speed with a mix solution of Mannitol and Tris-HCl, collecting 1 ml of homogenate, adding CaCl_2_, centrifuging at 9,000 × g for 10 min, removing the supernatant and centrifuging at 3,400 × g for 20 min. The pellet was resuspended in Tris–Hepesbuffer and used for enzymatic assays. Trypsin and amylase activities were assayed according to Holm *et al*.^89^ and Métais & Bieth^90^, respectively. Enzymes of the brush border membrane, alkaline phosphatase (AP) and aminopeptidase N were assayed according to Bessey *et al*.^91^ and Maroux *et al*.^92^, respectively. Proteins were determined according to the Bradford^93^ procedure. Enzyme activities were expressed in milliunits of specific activity (i.e. mU mg protein^−1^) and units of total activity (i.e. U segment^−1^).

### Determination of kinetic of stomach pH following ingestion

On trial day 35 at 20 °C and 55 at 15 °C, fish were fasted for 48-h and then re-fed based on their treatment. Stomach pH was measured at the end of the fasting period, 30 min post feeding and then regularly during the digestion process (see Fig. S1). For each measurement, 8 individuals were randomly sampled within each replicate tank, anaesthetized with MS-222, and the stomach immediately removed. A pH electrode (WTW Inolab 720 pH meter, Xylem Analytics Germany, Weilheim, Germany) was then inserted and maintained in the anterior portion of the stomach. While dissections took place on ice, the pH was measured at room temperature and the electrode was calibrated every three measurements.

### Statistical analysis

Normality for SGR data was first assumed according to the central limit theorem and verified visually via a q-plot of the raw data and residuals. Differences in SGR, specific *ad libitum* food consumption rate and FCE were tested using two-way ANOVAs. The overall effect of temperature and *P*CO_2_ level on daily total food intake was examined using a two-way, nested ANOVA. Significant ANOVAs were followed by a Student-Newman-Keuls multiple comparison test to determine differences among experimental groups. At each temperature, a linear mixed-effects model (LME models) was used to predict changes in the mean and variance (and, thus, the dynamics) of stomach pH in juvenile sea bass according to ration and *P*CO_2_ treatments (reported in Table S1 and S2). Time was considered a random effect and *P*CO_2_ level and feeding level were fixed effects. At each temperatures, the predictions of the LME model for stomach pH were compared across treatments (*P*CO_2_ level, feeding level) and sampling times with an ANOVA followed by a Tukey test. Differences were considered significant at α = 0.05. Differences in the magnitude of the initial decline in stomach pH among the four treatment groups (2 feeding levels, 2 *P*CO_2_ levels) at each sampling time within temperature (e.g. after 3 or 8 hrs at 15 °C; 4 or 9 hrs at 20 °C) were assessed with a Kruskal-Wallis test. Differences were considered significant at α = 0.05. Enzymes activities (specific and total), of each enzymes, were first tested for a temperature effect via a one-way ANOVA. Differences between *P*CO_2_ and feeding treatments were masked by high response of activity found at 20 °C, so potential effects were test separately between the two temperatures using two-way ANOVAs. Differences in enzyme activity were considered significant at α = 0.01. All statistical analyses were performed with R (ver.3.3.3; R Development Core Team).

## Supplementary information

Supplementary Material.
