# Supplementary material for: Food availability modulates the combined effects of ocean acidification and warming on fish growth
Source: Sci Rep. 2020 Feb 11;10:2338. doi: 10.1038/s41598-020-58846-2 (PMC7012865; doi:10.1038/s41598-020-58846-2)
Supplement: Supplementary file 1 — Supplementary Material. [file 41598_2020_58846_MOESM1_ESM.docx]

**Food availability modulates the combined effects of ocean acidification and warming on fish growth**

Louise Cominassi^1 *^, Marta Moyano^1^, Guy Claireaux^2^, Sarah Howald^1, 3^, Felix C. Mark^3^, José-Luis Zambonino-Infante^4^, Myron A. Peck^1^

^1^ Institute of Marine Ecosystem and Fisheries Science, Center for Earth System Research and Sustainability (CEN), University of Hamburg, 22767 Hamburg, Germany

^2^ Université de Bretagne Occidentale, LEMAR (UMR 6539), Centre Ifremer de Bretagne, 29280 Plouzané, France

^3^ Alfred Wegener Institute Helmholtz Centre for Polar and Marine Research, Integrative Ecophysiology, 27570 Bremerhaven, Germany

^4^ Ifremer, LEMAR (UMR 6539), Laboratory of Adaptation, Reproduction and Nutrition of Fish, Centre Ifremer de Bretagne, 29280 Plouzané, France

*Corresponding author

[+49 40 42838-6653](tel:+49-40-42838-6653)

[louise.cominassi@uni-hamburg.de](mailto:louise.cominassi@uni-hamburg.de)

[louise.cominassi@gmail.com](mailto:louise.cominassi@gmail.com)

**Supplementary tables:**

**Table S1: Significance of terms for the linear mixed-effect model (LME model) on the impact of feeding and *P*CO_2_ levels on the dynamic of stomach pH in juveniles European sea bass reared at 15°C.** Abbreviation: DF, degrees of freedom.

|  | **Value** | **Standard Error** | **DF** | **t-value** | **p-value** |
| --- | --- | --- | --- | --- | --- |
| **Intercept** | 4.755315 | 0.3963488 | 183 | 11.997806 | 0.0000 |
| **Ration** | -0.353966 | 0.4967770 | 183 | -0.712526 | 0.4770 |
| ***P*CO_2_** | -0.000396 | 0.0003884 | 183 | -1.020551 | 0.3088 |
| **Time** | **0.020390** | **0.0080774** | **55** | **2.524326** | **0.0145** |
| **Ration : *P*CO_2_** | -0.013210 | 0.0100713 | 183 | -1.311601 | 0.1913 |
| ***P*CO_2_ : Time** | 0.000002 | 0.0000079 | 183 | 0.235830 | 0.8138 |
| **Ration : Time** | 0.000389 | 0.0005559 | 183 | 0.699277 | 0.4853 |
| **Ration : *P*CO_2_ : Time** | -0.000008 | 0.0000113 | 183 | -0.687857 | 0.4924 |

**Table S2: Significance of terms for the linear mixed-effect model (LME model) on the impact of feeding and *P*CO_2_ levels on the dynamic of stomach pH in juveniles European sea bass reared at 20°C.** Abbreviation: DF, degrees of freedom.

|  | **Value** | **Standard Error** | **DF** | **t-value** | **p-value** |
| --- | --- | --- | --- | --- | --- |
| **Intercept** | 4.242492 | 0.4961067 | 161 | 8.551571 | 0.0000 |
| **Ration** | -0.62783 | 0.6308207 | 161 | -0.099525 | 0.9208 |
| ***P*CO_2_** | 0.000657 | 0.0005002 | 161 | 1.312855 | 0.1911 |
| **Time** | **0.033503** | **0.0134417** | **47** | **2.492456** | **0.0163** |
| **Ration : *P*CO_2_** | -0.000493 | 0.0007062 | 161 | -0.697884 | 0.4863 |
| ***P*CO_2_ : Time** | -0.000013 | 0.0000138 | 161 | -0.965925 | 0.3355 |
| **Ration : Time** | 0.010245 | 0.0170718 | 161 | 0.600090 | 0.5493 |
| **Ration : *P*CO_2_ : Time** | -0.000011 | 0.0000193 | 161 | -0.583927 | 0.5601 |

**Supplementary figures :**

**
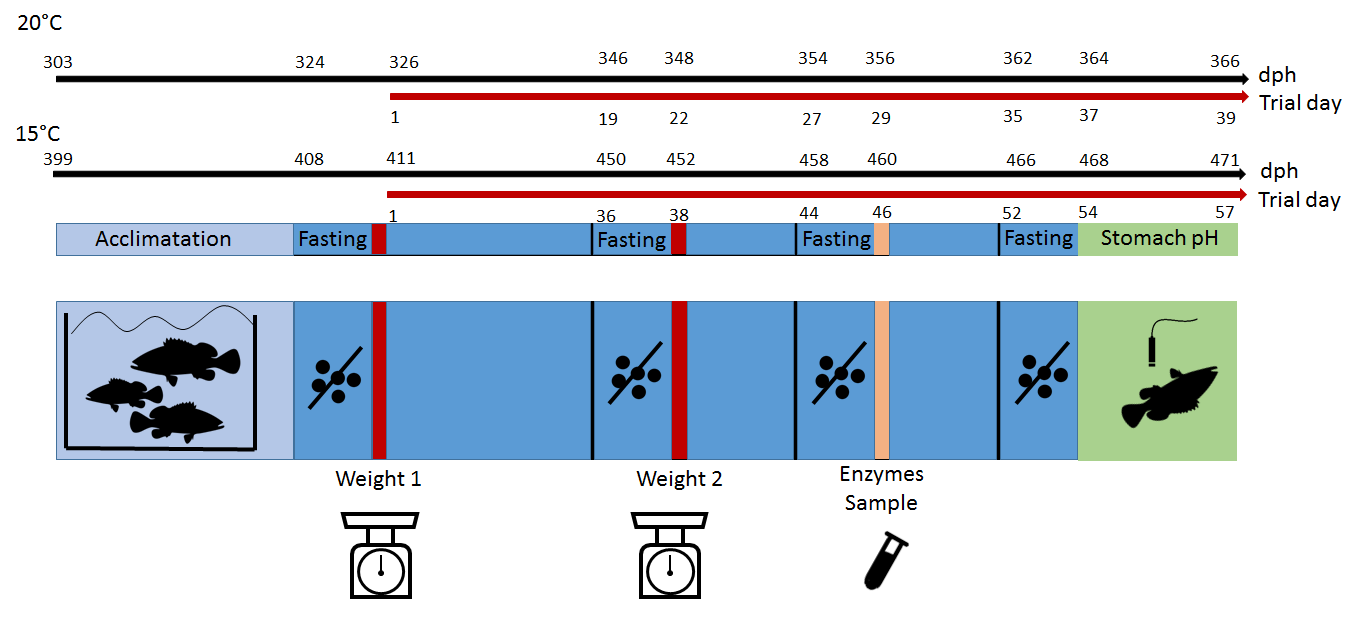
**

**Figure S1**: Proceeding of the feeding trial at two temperature regimes^94^. Dph: days post-hatch

Fish icons (CC) by Adam Zubin, MV.


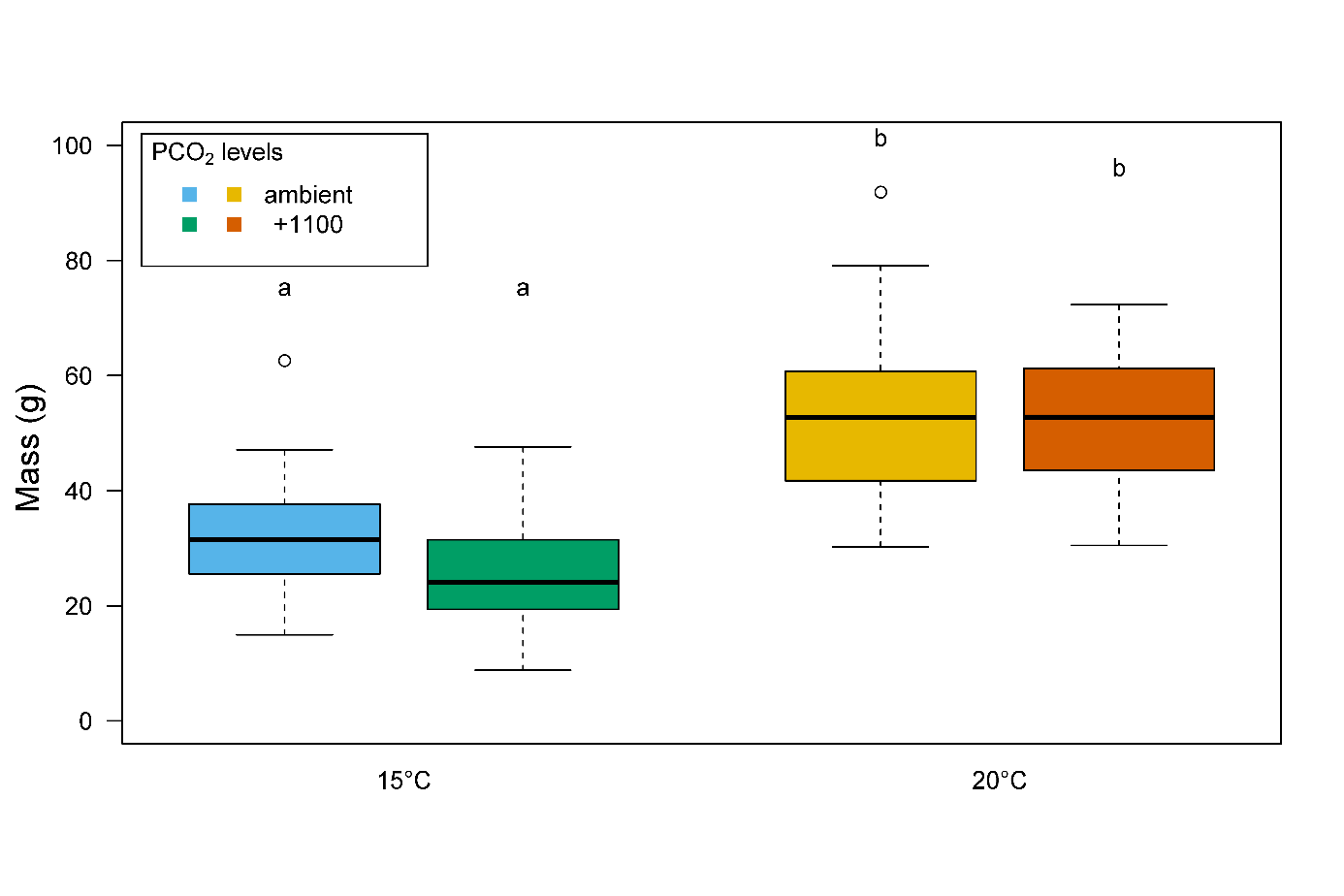


**Figure S2.** Box and whisker plots of wet mass of fish reared at two temperature regimes and two *P*CO_2_ levels at 367 dph and 277 dph at 15°C and 20°C, respectively^94^. The whiskers denote the 10^th^ and 90^th^ percentiles, the box denotes the 25^th^ and 75^th^ percentiles, the median value is shown (horizontal line) as well as outliers (points).


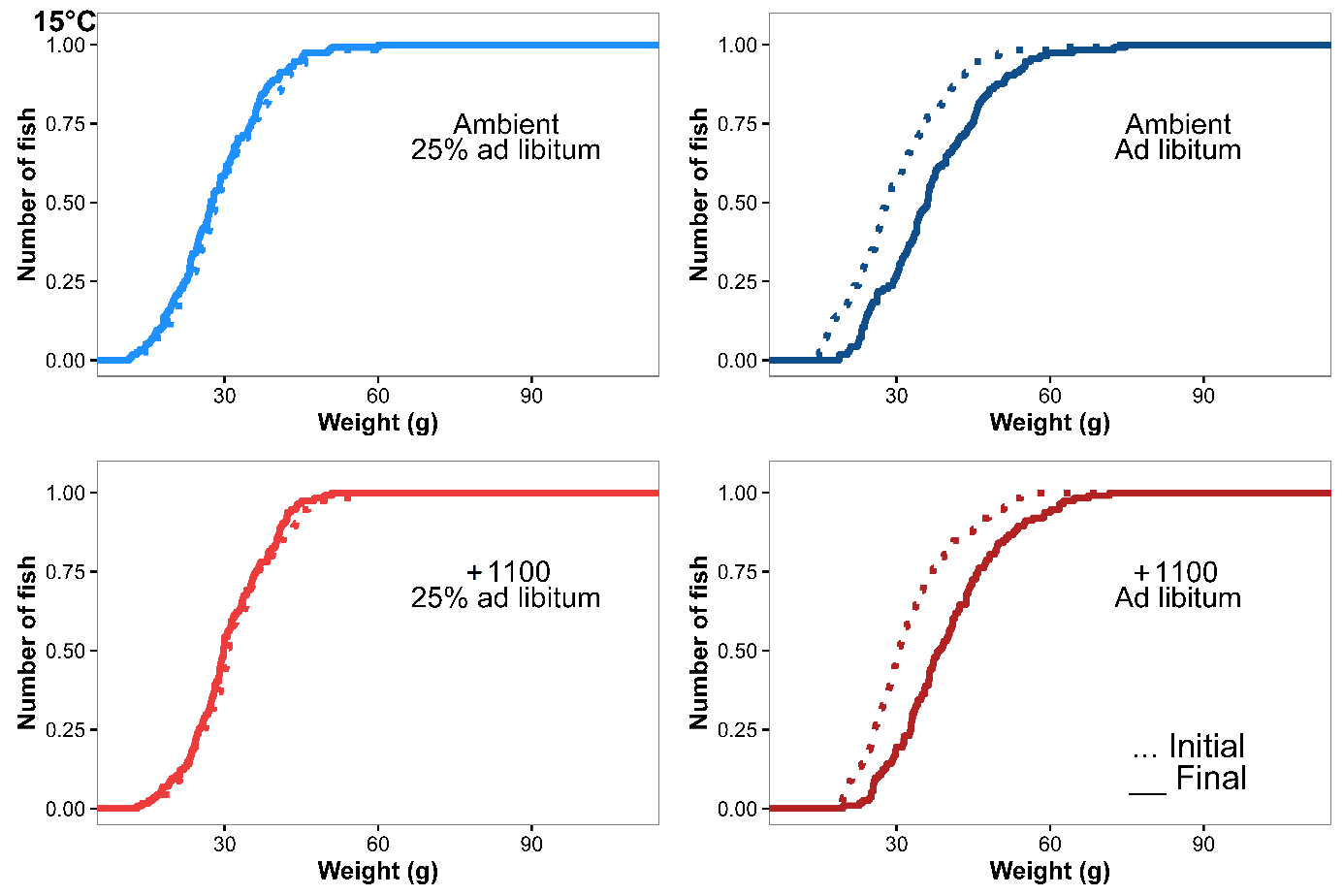


**Figure S3.** Wet mass cumulative distribution of fish reared at 15°C based on the initial and final measurements for each condition^94^.


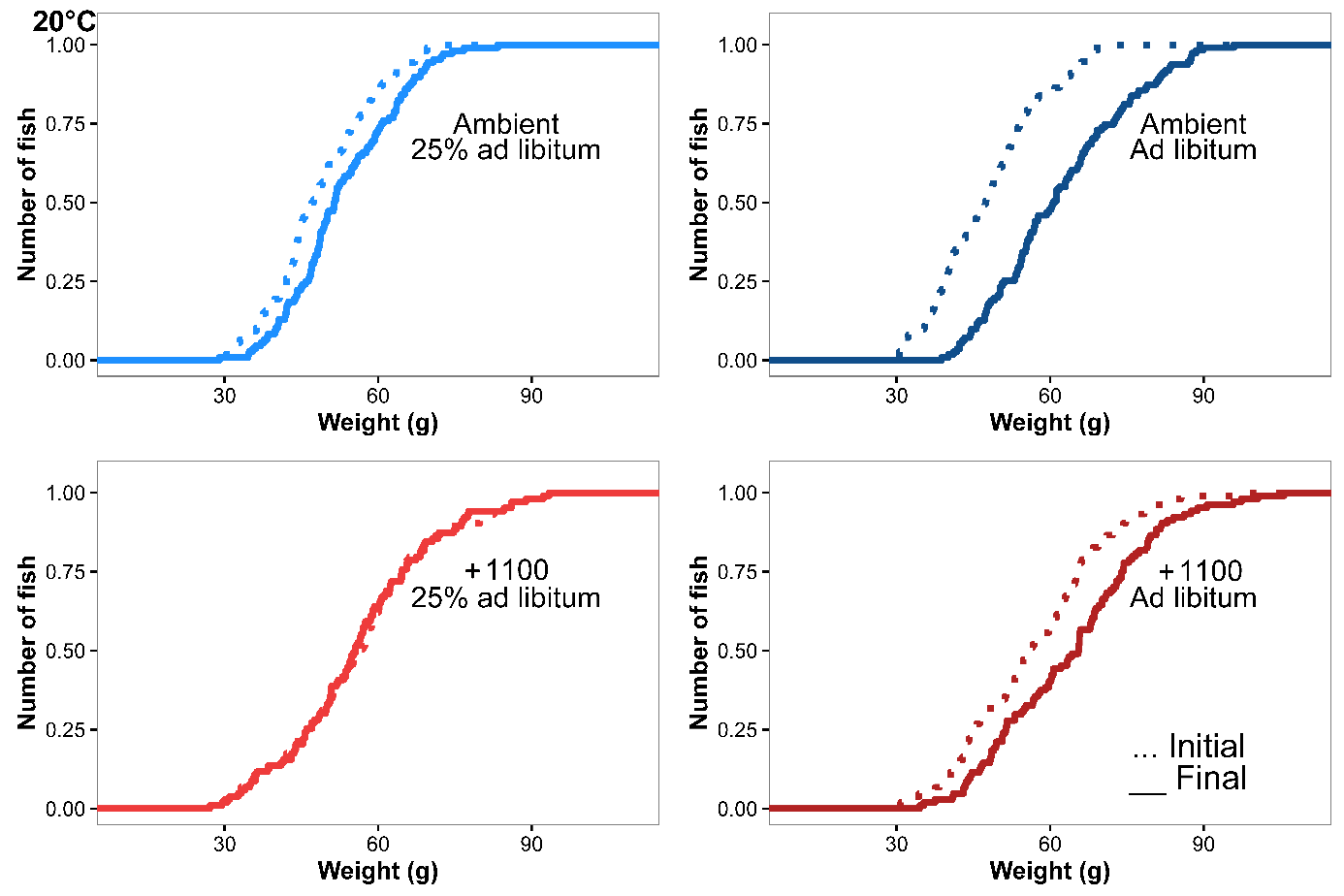


**Figure S4.** Wet mass cumulative distribution of fish reared at 20°C from the first weighing (Initial) to the second weighing (Final) for each condition^94^.


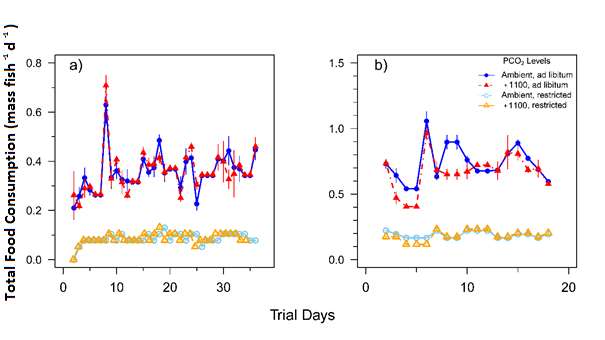


**Figure S5.** Mean (± SE, n=3) daily food consumption by juvenile sea bass at two *P*CO_2_ and two feeding levels during each of two feeding-growth trials (a) 15°C and b) 20°C) ^94^. In each trial, *P*CO_2_ levels were ambient (circles, 650 µatm) or +1100 (triangles, 1700 µatm) (see text) and feeding levels were *ad libitum* (filled symbols) or restricted (25% *ad libitum*, unfilled symbols).
